# Post-Graduate Opportunities in Special Hospitals

**Published:** 1906-09-01

**Authors:** 


### POST-GRADUATE OPPORTUNITIES IN SPECIAL HOSPITALS.

The Teaching Gift a Necessity.

Our friends of the great teaching medical schools in Scotland have ever kept a keen eye on the business side of medical teaching. They have long known that it pays to appoint to their staff only men of proved capacity for teaching. Not only must the successful candidate for positions in their universities possess high qualifications, but a *sine qua non* is that of power to impart knowledge with facility and aptness to others. Such being essential to success, as not every man can teach, we would strongly recommend the consideration of it to the authorities of our special teaching schools in London.

Abundant Clinical Material.

For many years after their establishment special hospitals had a protracted and arduous struggle for sheer existence, and their chief opponents were the general hospitals. The special hospitals were stigmatised as superfluous, and said neither to supply any want nor fulfil any rôle that was not amply provided at the general hospitals. The public gave, however, an emphatic and conclusive answer to this erroneous assertion as echoed in the busy hum of the overcrowded out-patient departments of most special institutions. They are now recognised as important and, indeed, invaluable factors in the struggle against disease.

Just as these charitable centres had to contend against much strenuous opposition before being acknowledged as essential in the amelioration of human suffering, so at present their claim to be admitted as indispensable for clinical teaching, though disputed, is being firmly maintained. During the last few years a most laudable change has come over the mind of the average medical graduate. Generally after short, practical experience in actual medical and surgical work a firm conviction of his many and great shortcomings has possessed him. The immediate result of this feeling is that he returns to drink again at the fountains of knowledge, and finds his cravings most satisfactorily gratified at many of the special hospitals.

If the ambition of many of the special teachers—that they should give the best response to the cry of the post-graduate for special instruction—is not realised, then the fault will assuredly lie with the special hospitals themselves, because the anxious attentive audience of students is certain, and the clinical material is, if anything, over-abundant in amount, and undoubtedly unrivalled in quality.

There is no department of special study that the medical graduate can with greater advantage give some time to become practically familiar with than that of the affections of the nose, throat, and ear. It is simply astonishing to note the number of patients sent by practitioners to special hospitals. The out-patient department is the place to become familiar with those difficulties in diagnosis which so frequently call for appeal from the ordinary medical man. It is most instructive to notice at the hospital where and how failure is explained.

Advantage of Special Knowledge.

Very often, for instance, patients suffering from intractable cough and hoarseness have been put through the whole range of pharmacopœial sedative expectorants, and the cause is only located and successfully treated in the nasal cavity. Again, a dyspepsia that may have baffled the best efforts of the experienced practitioner for many a long day may be quickly traced to an empyema of one or more of the sinuses of the head and the constant swallowing of septic material. Headache, very frequently after much and varied medical treatment, is finally found to be induced by nasal obstruction due to hypertrophic turbinated bones, which had not been considered at all as a causative condition. It is the bounden duty of the medical man to become able at least to diagnose many of these conditions, so that he may save his patients much prolonged suffering, and from drifting on until a serious surgical operation becomes imperative.

### The Ophthalmoscope.

Perhaps there is nothing so much desired by the earnest practitioner who has for years been immersed in the demands of busy practice as the opportunity to return for a time to his old school in order to gain a practical knowledge of modern methods of diagnosis and treatment. Among these methods the use of the ophthalmoscope may be said to be particularly attractive. Recent years have diffused the conviction of the value of ophthalmoscopic examination, not merely in special, but also in general, practice. And, indeed, it may be said that this method is fully as important in general medical work as it is in ophthalmic surgery. Hence it arises that many practitioners whose student days reach back some ten or fifteen years, when little regard was paid to the ophthalmoscope outside the eye department, now find themselves anxious to gain some experience of this instrument. A resolute man can do a good deal even among the opportunities of his own practice, but the initial difficulties can be much minimised by an experienced and sympathetic teacher. And nowadays there are numerous and excellent chances offered even to those who can give but little time to the work.

### Special Opportunities in London.

In London especially such opportunities are particularly numerous. First must be mentioned the Royal London Ophthalmic Hospital, City Road, E.C., still popularly spoken of under its old name of "Moorfields." Here is a wealth of clinical material probably unequalled at any hospital in the world. The clinic commences every morning at nine o'clock, and often extends well into the afternoon. Associated with each surgeon are one or more experienced clinical assistants, and these play an important part in the teaching scheme. Another excellent feature is to be found in the special classes where selected cases of all kinds of diseases are shown.

### Classes at "Moorfields."

These classes are conducted by the surgeons, who are in many instances men of the highest professional reputation, and form an excellent opportunity of becoming familiar both with the external diseases of the eyes and with ophthalmoscopic changes. Whilst recognising the predominant position of "Moorfields," the smaller hospitals and schools must be allowed to have some advantages.

### Some Advantages in Smaller Hospitals.

They are much less crowded, both with patients and students. Hence there is not the same necessity to hasten the work, and more attention may be given to the practitioner intent on gaining knowledge. Here, also, there are frequent series of special class-demonstrations, where a great variety of the manifestations of disease may be seen in a limited space of time. Indeed, at any one of the following hospitals the practitioner may be sure of enjoying excellent advantages and of getting substantial help from the members of the hospital staff: The Central Ophthalmic Hospital, Gray's Inn Road, W.C.; the Royal Eye Hospital, St. George's Circus, S.E.; and the Royal Westminster Ophthalmic Hospital, King William Street, W.C. In each of these the fees charged are very modest, and full particulars can be obtained on application to the hospital secretary.

### Facilities at the Polyclinic.

It sometimes happens that the practitioner who is quite unfamiliar with the ophthalmoscope finds it difficult to make a start at any hospital, where, as can be readily understood, time does not permit of much attention to elementary instruction. In such circumstances it is advisable, if possible, to get some preliminary direction and practice, and a class for this purpose is conducted at the Polyclinic, 22 Chenies Street, W.C. The fee is one guinea.

### The Provinces.

Altogether London offers excellent facilities for those who wish to enrich their professional equipment by the addition to it of a knowledge of ophthalmology. In Scotland and the provinces the opportunities are also very good. There are special post-graduation courses in Edinburgh and Glasgow, and also in Manchester. A word must be said of the holiday course in ophthalmology which has been held at Oxford, in July, during the last two years. It extends over a fortnight, and the greater part of the day is occupied in practical work. Application should be made to Mr. R. W. Doyne, University Reader in Ophthalmology.

### Queen Charlotte's Lying-in Hospital, Marylebone Road, N.W.

Qualified Medical Practitioners attending this hospital are permitted to do so for four weeks for a fee of £8 8s. The residence is at No. 2 Cosway Street, N.W. Terms for board and residence £1 10s. weekly. The whole time must be devoted to the work; when sent for they must attend at once. While in the labour wards the graduates shall, in the absence of the resident medical officers, act under the directions of the sister-midwife. They will not be allowed to make a vaginal examination until they have disinfected their hands according to the prescribed regulations and to the satisfaction of the resident medical officers, or the sister-midwife-in charge. They shall always wear the overalls provided while in the labour wards. They shall not perform any obstetric operation without the permission of one of the physicians or resident medical officers.

### The Hospital for Diseases of the Skin, Blackfriars.

Gives practical courses of cutaneous histopathology and diagnosis and treatment of diseases of the skin and syphilis at frequent intervals. They are conducted by T. J. P. Hartigan, F.R.C.S. (Eng.). Apply, G. A. Richardson, Secretary.

National Hospital for the Paralysed and Epileptic, Queen Square, W.C.

This hospital is a School of Medicine of the University of London, and has been recognised by the conjoint Board for England as a place where part of the fifth year may be devoted to clinical work. The following arrangements have been made for the practical study of diseases of the nervous system by gentlemen who are students of qualified practitioners. The out-patient practice is open without fee from 2.30 every day (except Thursday and Saturday), when patients are seen by the physicians for out-patients, Drs. Tooth, James Taylor, Risien Russell, and Aldren Turner, and the assistant physicians, Drs. Batten and Collier. The in-patient practice is only open to those who take out the hospital course. The physicians for in-patients attend at 2.30 on the following days:—Sir W. Gowers on Mondays, Dr. Beevor on Fridays, Dr. Ferrier on Wednesdays, and Dr. Ormerod on Tuesdays. Three courses of lectures and clinical demonstrations will be given in each year:—Winter course in October, November, December. Spring course in January, February, March. Summer course in May, June, July. They will be given in the theatre, on Tuesdays and Fridays, at 3.30 p.m., by one of the members of the staff. The next course will begin in October. Surgical operations will be performed by Sir Victor Horsley and Mr. Ballance, at 9 a.m. on Tuesdays, of which notice will be posted in the hospital. The out-patient hospital practice and lectures are free, but for the hospital course a fee of two guineas for three months or three guineas for six months is charged. The museum is available for the prosecution of special work under the pathologist, for which a separate fee of two guineas for three months is charged. Apply C. E. Beevor, Dean of the Medical School.

The Hospital for Women, Soho.

In connection with the out-patient department there has been for some years a well-organised clinical department. Clinical assistants to the gynæcologists to out-patients are appointed. The appointments are open to qualified medical men. The hospital contains 60 beds. In the out-patient department there were over 4,000 new cases during the past year, the total number of out-patient attendances being 15,778. This large number affords opportunities for examining and studying many kinds of the diseases of women. Clinical assistants receive notice of all operations performed within the hospital, and every facility is afforded them in the out-patient department of obtaining experience in diagnosis and treatment and the practical use of instruments. Fee for one month, three guineas; for each subsequent month, two guineas and a half. A certificate is given at the end of a three-months' course. Any further information can be obtained by writing to the Dean at the hospital.

Central London Throat and Ear Hospital, Gray's Inn Road, W.C.

In addition to the in-patient department, which is undergoing enlargement, and will be opened in November, the hospital has a large out-patient clinique which is open to medical practitioners and students. Last year 354 in-patients were treated and 10,050 out-patients were seen, involving over 50,000 separate attendances. Operation days: In-patients and out-patients, Tuesday and Friday, at 2.30 p.m. Special courses of practical demonstrations are given weekly on Wednesdays during the winter and summer sessions by the members of the staff. They are so arranged that practitioners joining at any time are enabled to complete the group of subjects in a course of six weeks. The fee for this course is one guinea, or, with daily attendance at the out-patient department, two guineas. The fee for general clinical attendance is three guineas for three months and five guineas for six months.

All information relating to teaching arrangements can be obtained on application to the Dean, Dr. Wyatt Wingrave. Apply to Richard Kershaw, Secretary.

Hospital for Diseases of the Throat, Golden Square, London, W.

Clinical instruction in the diagnosis and treatment of disease is given daily in the out-patient department from 2.30 to 5 p.m., on Tuesdays and Fridays from 6.30 to 9 p.m., and on Mondays at 9.30 a.m. Major operations are performed on Tuesday, Wednesday, Thursday, and Friday mornings and on Friday afternoons. Minor operations are performed daily (Mondays excepted) at 9.30 a.m.

Practitioners and medical students are admitted to the practice of the hospital at a fee of five guineas for three months, seven guineas for six months, or ten guineas for perpetual studentship. Apply to Charles A. Parker, Dean.

